# Camptocormia in Parkinson's Disease: A Muscle Disease Due to Dysregulated Proprioceptive Polysynaptic Reflex Arch

**DOI:** 10.3389/fnagi.2016.00128

**Published:** 2016-06-21

**Authors:** Walter J. Schulz-Schaeffer

**Affiliations:** Prion and Dementia Research Unit, Department of Neuropathology, University Medical Center GöttingenGöttingen, Germany

**Keywords:** camptocormia, proprioception, kinesthesia, golgi tendon organ, myopathy

Camptocormia (from the Greek “kamptein” = to bend and “kormos” = trunk) is an anterior flexion of the thoracolumbar spine while standing, walking, or sitting that disappears in the supine position. The syndrome, also known as “bent spine syndrome,” occurs in nearly 10% of idiopathic Parkinson's disease (iPD) patients (Yoritaka et al., 2013), but also in other neurodegenerative diseases and may occur in myopathies with axial involvement, in all forms of myositis, in dystonia, as a pharmacological side effect or as functional disorder. It causes marked impairment in quality of life and often leads to social isolation. The pathophysiology of camptocormia is as heterogenous as the causes of underlying diseases are, but recent work has provided some insights into the pathophysiology of PD-associated camptocormia and opened options for treatement. These findings show, how important it is to understand the interaction between muscle innervation, Golgi tendon organ and the central nervous system in regulating muscle tone.

## Dystonia vs. myopathy

Although, the bent spine syndrome has been known since first descriptions by Earle (1815) and appears in the original description of James Parkinson's case series in 1817 (Parkinson, 2002), the association of camptocormia with Parkinson's disease was definitively described by Djaldetti et al. (1999). Subsequently several small case series including muscle biopsies have been published, and myositis, mitochondrial disturbances, dystonia, or myopathy have been discussed as etiologies of the syndrome.

In the beginning of this century, larger series of paraspinal muscle biopsies in iPD patiens suffering from camptocormia revealed myopathic changes (Margraf et al., 2010; Spuler et al., 2010). Subsequently it was possible to exclude a mitochondriopathy and to define the myopathological changes that were common in all iPD camptocormia patients. By comparing samples of paraspinal and deltoid (a common biopsy muscle of the limbs) muscles of myopathologically healthy autopsy controls, a higher physiological content of mitochondria and so called “ragged red fibers” in the paraspinal than in the limb muscles was observed (Wrede et al., 2012), showing that mitochondrial content in muscle fibers seems to depend on dedication, force and function of muscles. By comparing the mitochondrial pattern in paraspinal muscles of 14 camptocormia biopsies and 10 autopsy controls, a mitochondriopathy in camptocormia of iPD patients could be ruled out (Wrede et al., 2012).

Interestingly, in the paraspinal muscles of all iPD camptocormia patients a reduction in type-2 fibers were observed, as well as a marked increase in size of type-1 fibers, an increase in connective tissue and fatty degeneration within muscle fascicles, and frequently defects located centrally in muscle fibers, comprising a loss of oxidative enzymes, visible in NADH-TR, SDH, COX, and MAG reactions. Within these defects, an increase of acid phosphatase reactivity was observed. Ultrastructurally, the defects showed a disarrangement of contractile elements and rod-like structures (Wrede et al., 2012). A myositis was not detectable. The lesions are different from central core diseases, although a ryanodine receptor 1 mutation can cause a camptocormia (Loseth et al., 2013). In core diseases, a type-1 fiber hypotrophy is seen and lesions lack acid phosphatase reactivity (Jungbluth, 2007).

The interpretation of these changes suggests a myopathy that could explain the loss of muscle strength in the paraspinal muscles as the main clinical symptome of camptocormia. Whereas all key aspects of the myopathological changes were detectable in all camptocormia muscle biopsies, the extent varied. Especially the extent of (reactive) fibrosis and (reactive) fatty degeneration of paraspinal muscles differed. The degree of fibrosis correlate with the severity of the syndrome (Wrede et al., 2012). Biopsies of muscles involved in dystonic movements have up to now revealed no histopathologically detectable myopathological changes (Swash and Fox, 1976). This may be because dystonic events result in transient muscle contraction, whereas clinical investigations in camptocormia suggest continuous muscle contraction. A hardening of paraspinal muscles is always found when iPD camptocormia patients are in an upright position and electromyographical recordings show continuously elevated firing (DiMatteo et al., 2011; Doherty et al., 2011; Tinazzi et al., 2013). Myopathologically, camptocormia is unlikely due to a dystonia, but the myopathological changes may be due to a secondary myopathy. Fibrosis and fatty changes are obviously secondary.

## Myopathological findings in camptocormia lead to the hypothesis of proprioceptive dysregulation

The myopathological findings in camptocormia show remarkable parallels to those that were found 40 years ago by Karpati et al. in experimental tenotomy of achilles tendons in rats. Experimental tenotomy leads to core-like lesions in the center of type-1 fibers, which show an increase in acid phosphatase activity but not an increase in lysosomal structures (Karpati et al., 1972). Fibers of tenotomised muscles lack ATPase and SDH activity (Shafiq et al., 1969). Ultrastructurally, the core-like lesions have no normal register of sarcomeres. Sarcomeres in the lesions were disintegrated and Z-band streaming and electron-dense patches or plaques could be observed. In the lesions, mitochondria were reduced (Shafiq et al., 1969). These changes could not be observed in muscles that were tenotomized after denervation or after chordotomy (Karpati et al., 1972). Obviously, these experimental lesions developed only after interruption of the muscle tension input to the polysynaptic reflex arch, while the rest of the reflex arch was functionally intact. The description depicts the situation of a proprioceptive dysregulation.

The lesions described in tenotomy experiments are strikingly similar to the lesions observed in paraspinal muscles of camptocormia patients. These similarities point to the likelihood that camptocormia may also be related to a proprioceptive dysregulation. Other than in experimental tenotomy, the lesion site in camptocormia is most likely not at the level of the muscles. The association of camptocormia with Parkinson's disease suggests that the lesion is at the level of the central nervous system. Muscle tone regulation of paraspinal muscles is different from that of limb muscles (Gurfinkel et al., 2006). The body mass is anterior to the spinal column and the function of axial (i.e., paraspinal) muscles is to compensate any head or limb movements immediately to stabilize the trunk. Whereas, in limb muscles a shortening of the muscle length is associated with an increase in muscle tone, in paraspinal muscles the tonic level needs to increase in stretched muscles in order to stabilize the axis (Wright et al., 2007). The basal ganglia are involved in the control of postural muscle tone via proprioception (Takakusaki et al., 2004; Konczak et al., 2009). In PD, axial muscles are hypertonic and the hypertonicity correlates with UPDRS (unified Parkinson disease rating scale) scores (Wright et al., 2007). A deficit in the integration of proprioceptive information in postural control has been shown in PD (Vaugoyeau et al., 2011; Mongeon et al., 2015). It is possible that camptocormia in PD is an extreme form of muscle tone dysregulation.

## Parallels between camptocormia and tendon rupture seen with imaging methods

Clinical presentation and MR images of the paraspinal muscles in camptocormia show remarkable parallels with the cramp-like pain and radiological alterations seen with torn tendon muscles. In camptocormia, the syndrome presents with serious back pain and a marked, well-palpable hardening of paraspinal muscles (Margraf et al., 2010). MR imaging shows a hyperintense signal of paraspinal muscles in the STIR sequence and a signal decrease in T1-weighted images, indicating edema and swelling. In other camptocormia patients, however, an increase in signal intensity in T1-weighted images has been observed. This is interpreted as fatty degeneration and muscle atrophy. Interestingly, these changes are associated with the length of time that patients suffered from camptocormia (Nakane et al., 2015). Whereas, edema and swelling are observed in the first 2 years of the syndrome, fatty degeneration and atrophy are observed later in the disease course (Margraf et al., 2015). With torn tendons, an initial painful contraction in the respective muscles can be observed, as for example in “Popeye syndrome,” when the biceps is involved (Delle Rose et al., 2012). Subsequently, structural alterations in the muscle and muscle shrinkage will take place. These alterations can be visualized by CT and MR imaging. The Goutallier classification in radiology provides stages of deterioration (Goutallier et al., 1994). Higher stages (more advanced deterioration) were explained by progressing fibrosis and fatty degeneration of the involved muscles (Gerber et al., 2009; Hoffmann et al., 2011).

## Other reasons for myopathological alterations in paraspinal muscles that resemble those of camptocormia

**Figure 1:**
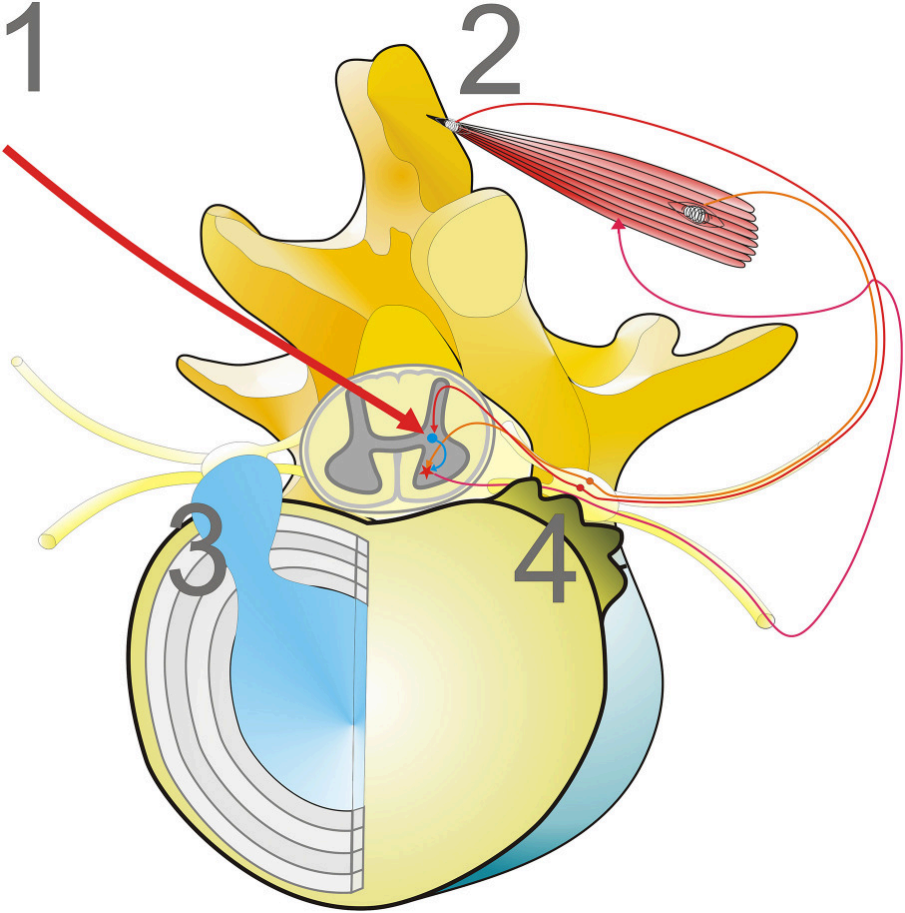
**Mechanisms disturbing the proprioceptive polysynaptic reflex arch can cause characteristic myopathological lesions**. (1) Central dysregulation, frequently associated with basal ganglia diseases, i.e., iPD; (2) Loss of muscle tension input to the polysynaptic reflex arch, while the reflex arch is functionally intact (i.e., tenotomy; Popeye syndrome); (3, 4) Mechanical irritation of dorsal root ganglia or dorsal root nerves, for example by disc herniation (3) or spondylophytes of disc plates (4) that occur as a phenomenon of aging.

The pattern of acid phosphatase-reactive myofibrillar disarrangement of paraspinal muscles is not limited to camptocormia. Similar myopathological changes may be observed in disc herniation, scoliosis, or in aged individuals (Mattila et al., 1986; Wharton et al., 1996), but not all these patients develop camptocormia-like myopathological changes (Ford et al., 1983). In general, the fibrosis is less severe in patients suffering from disk herniation than in camptocormia patients (Delisle et al., 1993). It is most likely that the myofibrillar disarrangement of paraspinal muscles can be induced by compression of nerve root fibers or dorsal root ganglia. Causes of compression may be herniation of an intervertebral disc or bony changes such as spondylophytes of vertebrae, a phenomenon that is frequently observed in older persons, or malformation of vertebrae. It seems to be irrelevant for the development of the characteristic myopathological changes, whether the disturbances of the proprioceptive polysynaptic reflex arch occur at the level of the Golgi tendon organ (tension receptor), the dorsal root ganglia (afferent fibers of tendon organs and muscle spindles), or of the central control. A synopsis of the different pathways is provided in Figure 1.

## Effect of STN neurostimulation on camptocormia in PD suggests central modulation of proprioception

The hypothesis that defects in the central control of proprioceptive function underlie the pathophysiology of camptocormia has led to the notion that deep brain stimulation (DBS) of the subthalamic nucleus (STN) may have a beneficial effect on camptocormia in iPD. STN-DBS is known to partially remove disturbances in proprioception (Maschke et al., 2005). Unfortunately, the literature report that about half of iPD patients with camptocormia who underwent STN-DBS showed no improvement in bent back angle (Chieng et al., 2015; Srivanitchapoom and Hallett, 2016). A recent retrospective study investigated whether DBS of the subthalamic nucleus would in principle be able to relieve bent back in camptocormia of iPD patients and which factors were correlated with the outcome (Schulz-Schaeffer et al., 2015). Twenty-five iPD patients suffering from camptocormia who underwent DBS of the STN were given a standardized questionnaire. Information from medical records and family members were used additionally. Thirteen patients were classified as responders who showed improvement in the bending angle of the spine after STN-DBS of at least 50%; 12 were classified as non-responders. Responders and non-responders did not differ statistically with regard to the male-to-female ratio, age at PD onset, period of PD before camptocormia, bending angle before DBS, UPDRS III before DBS or levodopa-equivalent dose before DBS. The positive predictive factor related to an improvement in the angle of bent back using STN-DBS was a short duration of camptocormia symptoms. All patients with a captocormia duration of up to 20 months improved, whereas all but one with camptocormia duration of over 40 months did not. A scar-like mechanism of fibrosis and fatty degeneration in long-term diseased paraspinal muscles may hinder the effect of DBS on these muscles.

## Conclusion

The polysynaptic reflex arch of the spinal cord integrates sensory information to motoric output. The sensory input comes from Golgi tendon organs, muscle spindles and joint receptors. Their information is not only integrated to motoric output, but may raise awareness such that motoric output can be influenced voluntarily. The sense of joint position, of movement (kinesthesia) and the sense of muscle strength are part of our self-awareness or proprioception. It is known that proprioception is impaired in some diseases of the central nervous system, for example in Parkinson's disease. Disturbances in proprioception—regardless of whether they originate centrally through neurodegenerative diseases or peripherally due to mainly age-related alterations—may cause characteristic myopathological changes in the axial musculature responsible for maintaining an upright body position, in extreme cases resulting in the syndrome known as camptocormia. Thus disturbances in the proprioceptive polysynaptic reflex arch may reflect mechanisms of neurodegeneration or mechanisms of aging.

## Author contributions

The author confirms being the sole contributor of this work and approved it for publication.

### Conflict of interest statement

The author declares that the research was conducted in the absence of any commercial or financial relationships that could be construed as a potential conflict of interest.
